# Effects of Platelet-Rich Plasma Injections on Periodontal Health During Accelerated Orthodontic Tooth Movement

**DOI:** 10.7759/cureus.69498

**Published:** 2024-09-16

**Authors:** May Akel, Basima Yosef, Fadi Khalil

**Affiliations:** 1 Department of Orthodontics, Faculty of Dentistry, Tishreen University, Latakia, SYR; 2 Department of Histopathology, Faculty of Dentistry, Tishreen University, Latakia, SYR

**Keywords:** accelerated orthodontics, acceleration, aspartate aminotransferase (ast), platelet-rich plasma (prp), tooth movement

## Abstract

Background: The interplay between orthodontics and periodontics is crucial for successful orthodontic treatment. Accelerating orthodontic tooth movement (OTM) can enhance treatment efficiency, but it is important to assess the impact of such methods on periodontal health. Platelet-rich plasma (PRP) has emerged as a promising adjunct in accelerating OTM, but its effects on periodontal health require further investigation.

Objective: This study aimed to evaluate the impact of PRP injections on periodontal health during accelerated OTM by comparing clinical and biochemical parameters between a PRP-enhanced orthodontic treatment group and a conventional orthodontic treatment group.

Methods: In this randomized controlled trial, 40 participants with anterior teeth crowding were divided into two groups: the PRP group (n = 20), which received standard orthodontic treatment with PRP injections, and the control group (n = 20), which received standard orthodontic treatment alone. Periodontal parameters, including periodontal probing depth (PPD), clinical attachment level (CAL), bleeding on probing (BOP), and gingival recession (GR), were recorded at baseline, four, eight, and 12 weeks. Salivary aspartate aminotransferase (AST) levels were measured at baseline, one hour, 24 hours, seven days, and 14 days post treatment.

Results: No statistically significant differences were found between the PRP and control groups regarding periodontal parameters (PPD, CAL, BOP, and GR) and AST enzyme levels throughout the study period. Both groups exhibited slight increases in PPD, CAL, GR, and BOP over time, consistent with typical orthodontic effects. AST levels showed fluctuations but no significant differences between the groups.

Conclusion: PRP injections did not demonstrate significant advantages over conventional orthodontic treatment in terms of periodontal health or AST enzyme levels. This suggests that PRP is a safe and non-inferior method for accelerating OTM without adversely affecting periodontal tissues. Further research with larger sample sizes and longer follow-ups is recommended to validate these findings and explore the long-term effects of PRP.

## Introduction

The interrelationship between periodontics and orthodontics has been extensively studied over the years. Orthodontic treatment can enhance oral hygiene by correcting dental irregularities and reducing or eliminating occlusal trauma. However, the placement of orthodontic appliances often leads to changes in oral hygiene habits and affects periodontal health [1]. Orthodontic forces trigger an acute inflammatory response, which reduces blood flow within the periodontal ligament and results in the release of inflammatory mediators. These mediators activate osteoclasts and osteoblasts: osteoclasts are responsible for bone resorption, while osteoblasts facilitate new bone formation. The activity of these cells can be monitored through biological indicators present in gingival crevicular fluid [2].

In modern orthodontics, there is a notable shift toward accelerating orthodontic tooth movement (OTM) to shorten treatment duration and mitigate the adverse effects associated with prolonged therapy [3]. Various methods for accelerated OTM have been proposed, including both surgical and non-surgical techniques. Surgical approaches include periodontally assisted accelerated orthodontics [4], corticotomy [5], piezocision, dentoalveolar distraction, corticision, and discision [6]. Non-surgical methods encompass micro-osteoperforations, micropulse, cyclic forces, resonance vibration, pulsed magnetic field forces, low-intensity laser therapy (LILT) [7], low-level light therapy (LLLT), low-level pulsed ultrasound, gene therapy, and the systemic and local administration of chemical agents [8]. Among the most promising local agents for accelerating OTM is platelet-rich plasma (PRP) [9]. PRP is an autologous plasma preparation enriched with a higher concentration of platelets compared to whole blood. Recent studies have highlighted the positive effects of PRP on accelerating OTM, with local injections around treated teeth resulting in faster tooth movement in animal models [10,11]

Numerous studies have examined the impact of orthodontic treatment on periodontal health. Fixed orthodontic appliances are often associated with gingival inflammation, primarily due to difficulties in maintaining oral hygiene and increased bacterial plaque accumulation [12-14]. Although some studies report a higher incidence of periodontal attachment loss in patients undergoing fixed orthodontic treatment compared to controls [15,16], most longitudinal studies indicate that gingival changes induced by orthodontic appliances are transient and do not cause permanent damage to periodontal structure [12-14].

Aspartate aminotransferase (AST) is a biological marker of tooth movement, released into the gingival crevicular fluid (GCF). AST, an enzyme predominantly found in the liver and heart, is typically located in the cytoplasm and is released during cellular apoptosis. Trauma to periodontal tissue stimulates AST release into the GCF. AST activity has been used as an indicator for bone remodeling and periodontal disease progression [17,18]. Previous research has shown increased AST activity in the GCF of patients with periodontal disease and elevated AST levels in saliva [19]. Several orthodontic studies have also confirmed increased AST activity in the GCF due to orthodontic forces [20,21].

The aim of this study is to evaluate the impact of utilizing PRP injections for accelerating OTM on periodontal health using clinical and immunological parameters.

## Materials and methods

Study design and sample size calculation

This randomized controlled clinical trial was conducted at the Department of Orthodontics, Faculty of Dentistry, University of Tishreen, from January 2022 to August 2023, following the Consolidated Standards of Reporting Trials (CONSORT) reporting guidelines. Ethical approval was obtained from the Scientific Research Council of Tishreen University (approval number: 2608), and informed consent was secured from all participants. This study was registered at the International Standard Randomised Controlled Trial Number registry under the number ISRCTN18306557.

The sample size was calculated using G*Power software (version 3.1.9.7; Heinrich Heine University Düsseldorf, Düsseldorf, Germany). Based on an effect size of 1.118, indicating a clinically significant 10% expected difference in tooth movement between groups, and the highest reported standard deviation of 0.54 [10], a sample size of 28 patients (14 per group) was determined to achieve a type I error rate of 5% and a power of 80%. To account for a potential 20% dropout rate, 40 patients were recruited (20 per group).

Inclusion and exclusion criteria

Eligible participants were individuals aged 16 to 25 years with anterior teeth crowding ranging from 3 to 6 mm, maintaining excellent oral hygiene evident by low plaque index and periodontal health, and without prior orthodontic treatment history. Exclusion criteria included systemic medical conditions, dental anomalies in tooth size or shape, and current use of anti-inflammatory medications.

Patients’ recruitment, randomization, allocation, and blinding

Out of 57 assessed patients, 40 met the inclusion criteria and were randomized into two equal groups. The first group received orthodontic therapy with PRP injections, while the second group received standard orthodontic treatment without supplementary interventions. Randomization was achieved using sealed envelopes containing the allocation sequence, ensuring that treatment assignments remained concealed from both researchers and participants until the designated phase of the study. The randomization sequence was generated by an independent academic specialist. Due to the nature of the interventions, blinding was not feasible for practitioners or patients; therefore, blinding was limited to outcome assessors only.

Study methods

Comprehensive pre-treatment records were collected, and orthodontic treatment began with the application of 0.022-inch brackets using the McLaughlin, Bennett, and Trevisi (MBT) technique (IOS, Boston, USA). Treatment continued until a 0.017 x 0.025-inch stainless steel archwire was in place.

**Figure 1:**
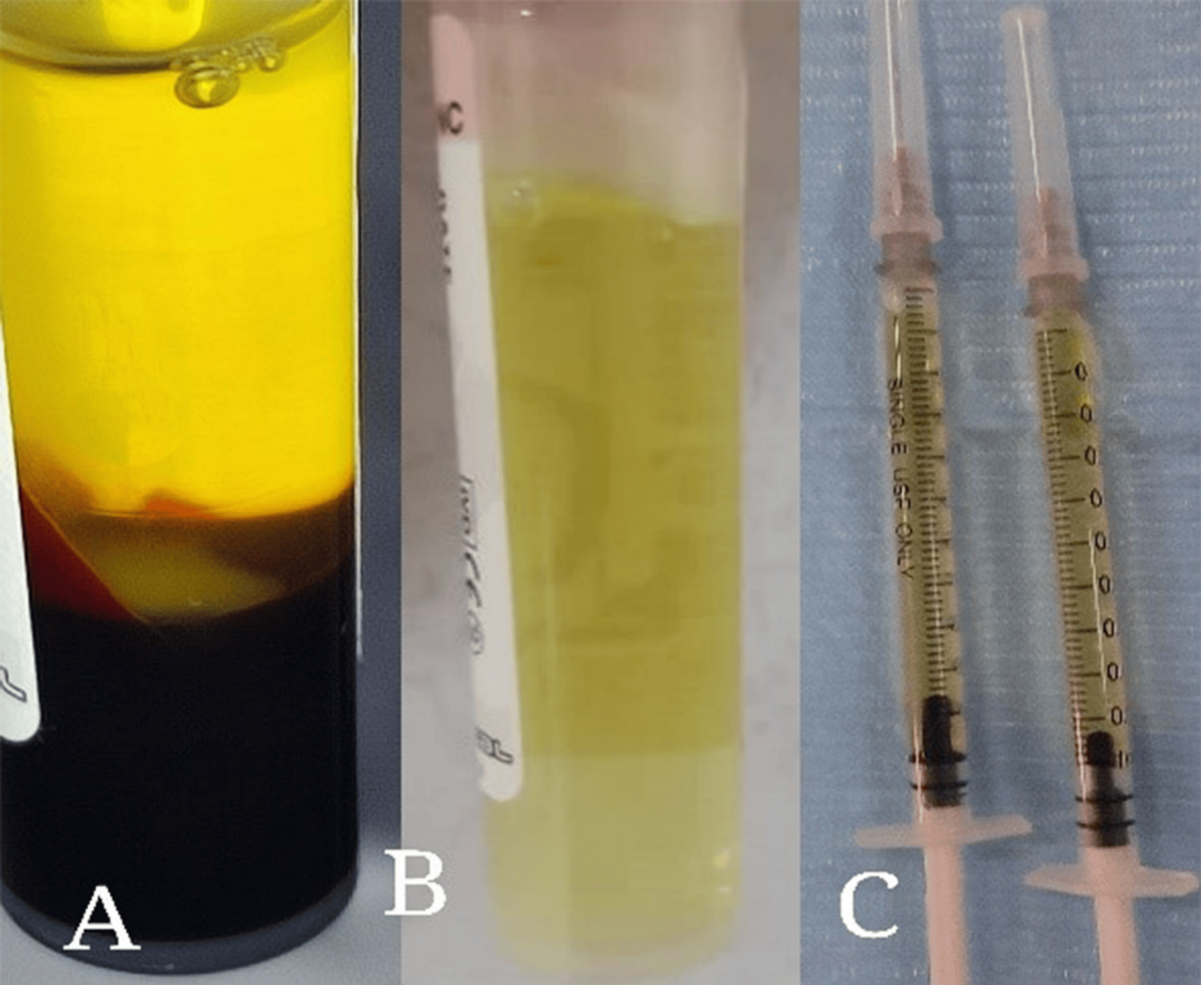
Platelet-rich plasma (PRP) preparation using the double-spin method. (A) Blood centrifuged after the first spin. (B) PRP formed after the second spin. (C) PRP ready to be used.

**Figure 2:**
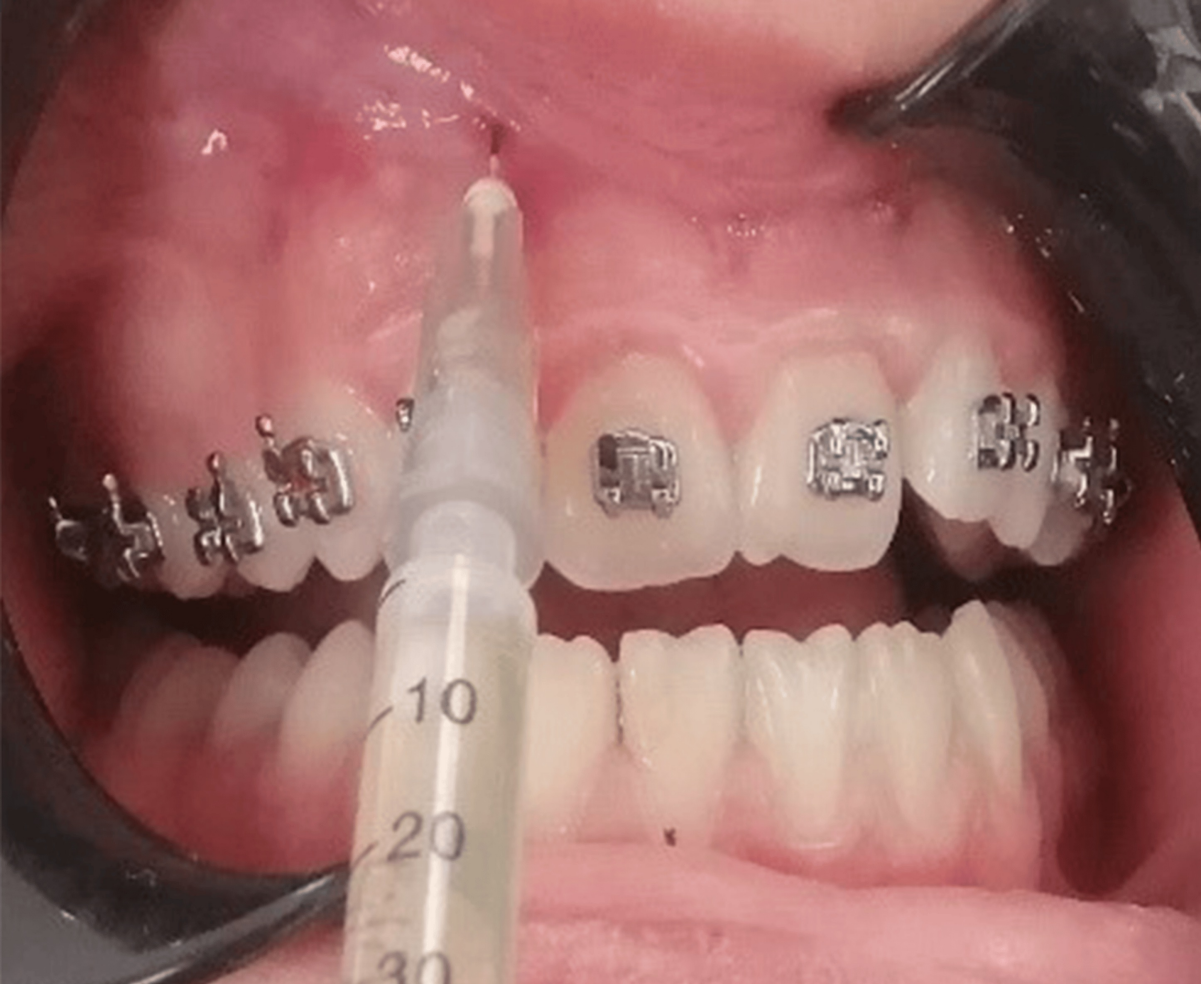
Injection of platelet-rich plasma on the buccal side.

In the PRP group, PRP was prepared using the double-spin method as described by Marx and Garg and Rashid et al. [10]. Buccal infiltration anesthesia was administered with 2% lidocaine hydrochloride containing 1:80,000 epinephrine (Kwang Myung Pharm, Seoul, Korea). A 0.5 ml aliquot of PRP was injected at four sites near the mesial and distal root surfaces of the lateral incisor on the buccal side (Figures 1, 2). The PRP was introduced submucosally, similar to the technique used for local anesthesia. Each patient received a total of 2 ml of PRP, with 0.5 ml injected through the attached gingiva. PRP injections were administered at zero, seven, and 14 days.

The control group received only the standard orthodontic treatment described earlier, without additional interventions. This group served as a baseline to compare the effectiveness of PRP in accelerating OTM.

Saliva samples were collected from all participants at baseline (before treatment), one hour after the start of treatment, one week after, and two weeks after treatment. Samples were stored at -80°C until analysis. Salivary AST activity was measured using a spectrophotometer. Reagent 1 included 1000 µL of Tris buffer (pH 7.65, 80 mmol/L), L-aspartate (240 mmol/L), malate dehydrogenase (MDH) (≥600 U/L), and lactate dehydrogenase (LDH) (≥900 U/L), incubated at 37°C for one minute. Reagent 2, containing 2-oxoglutarate and nicotinamide adenine dinucleotide (NAD) + hydrogen (NADH), was also incubated at 37°C for one minute. A 100 µL sample was then added for activity measurement. Periodontal health parameters, including periodontal probing depth (PPD), clinical attachment level (CAL), bleeding on probing (BOP), and gingival recession (GR), were recorded at baseline, four weeks, eight weeks, and 12 weeks.

Statistical analysis

Continuous measurements were reported as means and standard deviations and compared using two-sample t-tests and one-way ANOVA. Categorical variables were presented as percentages and compared using chi-square tests. Data normality was assessed with the Shapiro-Wilk test. A significance level of P < 0.05 was considered statistically significant. Statistical analyses were performed using SPSS® version 29.0.2.0 (IBM Corp., Armonk, NY).

## Results

Out of the initial 57 eligible patients, 17 were excluded: nine due to active periodontitis and eight due to systemic health issues. Therefore, 40 patients were included and received orthodontic treatment in the maxillary jaw. All participants completed the study successfully.

Demographic data

The mean age of the participants was 20.1 years, with the control group having a mean age of 19.6 years (range 16-20) and the PRP group having a mean age of 21.6 years (range = 20-23). Females comprised the majority (85.1%), with the PRP group having a higher proportion of females (100%) compared to the control group (90%). There were no statistically significant differences between the groups regarding age (P = 0.139) or gender (P = 0.107). Demographic characteristics are detailed in Table 1.

**Table 1:** Demographic characteristics of the included participants in each group. PRP: platelet-rich plasma; SD: standard deviation.

Groups	Age (mean ± SD)	P-value	Sex, N (%)		P-value
			Men	Female	
Control group (n = 20)	19.6 ± 2.3	0.139	0 (0%)	20 (100%)	0.107
PRP group (n = 20)	21.6 ± 1.9		2 (10%)	18 (90%)	

Periodontal parameters and level of AST enzyme at baseline

At baseline, the PRP group showed slightly higher values in PPD, CAL, and BOP compared to the control group. Conversely, the control group exhibited less GR than the PRP group. The AST enzyme level was lower in the PRP group. Statistical analyses using chi-square tests, independent t-tests, and one-way ANOVA indicated no significant differences between the two groups for PPD, CAL, BOP, GR, or AST levels at baseline (P > 0.05). Baseline values are summarized in Table 2.

**Table 2:** Periodontal parameters and level of AST enzyme at baseline. AST: aspartate aminotransferase; BOP: bleeding on probing; CAL: clinical attachment level; GR: gingival recession; PPD: periodontal probing depth; PRP: platelet-rich plasma; SD: standard deviation.

Variables	Mean (SD)		P-value
	Control group (n = 20)	PRP group (n = 20)	
PPD (mm)	2.53 (0.95)	2.87 (1.03)	0.291
CAL (mm)	2.79 (0.36)	3.07 (0.60)	0.317
BOP (%)	46.9 (0.46)	49.3 (0.44)	0.437
GR (mm)	0.66 (0.23)	0.87 (0.31)	0.051
AST (IU/L)	38.87 (25.20)	22.91 (21.78)	0.060

Changes in periodontal parameter measurements and AST enzyme levels

Both groups exhibited slight increases in PPD, CAL, and GR, and higher BOP percentages at four, eight, and 12 weeks compared to baseline. However, there were no statistically significant changes within or between the groups over time (P > 0.05). Detailed measurements of periodontal parameters during follow-up are presented in Table 3.

**Table 3:** Mean (standard deviation) of periodontal parameters at different follow-up periods. BOP: bleeding on probing; CAL: clinical attachment level; GR: gingival recession; PPD: periodontal probing depth; PRP: platelet-rich plasma.

Variables	After 4 weeks		After 8 weeks		After 12 weeks		P-value
	Control	PRP	Control	PRP	Control	PRP	
PPD (mm)	2.61 (0.98)	2.91 (0.98)	2.72 (0.91)	2.96 (0.93)	2.86 (0.87)	3.07 (0.90)	0.936
CAL (mm)	2.83 (0.67)	3.11 (0.83)	2.91 (0.62)	3.19 (0.76)	3.03 (0.58)	3.24 (0.72)	0.322
BOP (%)	49.7 (0.51)	51.1 (0.56)	51.9 (0.48)	52.6 (0.52)	53.1 (0.42)	53.9 (0.47)	0.536
GR (mm)	0.83 (0.31)	0.92 (0.39)	0.94 (0.27)	1.03 (0.32)	1.07 (0.24)	1.13 (0.28)	0.200
Variables	After 4 weeks		After 8 weeks		After 12 weeks		P-value
	Control	PRP	Control	PRP	Control	PRP	
PPD (mm)	2.61 (0.98)	2.91 (0.98)	2.72 (0.91)	2.96 (0.93)	2.86 (0.87)	3.07 (0.90)	0.936
CAL (mm)	2.83 (0.67)	3.11 (0.83)	2.91 (0.62)	3.19 (0.76)	3.03 (0.58)	3.24 (0.72)	0.322
BOP (%)	49.7 (0.51)	51.1 (0.56)	51.9 (0.48)	52.6 (0.52)	53.1 (0.42)	53.9 (0.47)	0.536
GR (mm)	0.83 (0.31)	0.92 (0.39)	0.94 (0.27)	1.03 (0.32)	1.07 (0.24)	1.13 (0.28)	0.200

AST enzyme levels in the control group increased after one hour, peaked at seven days, and then decreased by 14 days, with no statistically significant differences observed between the time points (P > 0.05). In the PRP group, AST levels initially increased after one hour, decreased at 24 hours, increased again at seven days, and decreased by 14 days, with the highest level recorded after one hour. One-way ANOVA indicated no significant differences between the groups or across time points (P > 0.05). Changes in AST enzyme levels during follow-up are summarized in Table 4.

**Table 4:** Mean values of AST enzyme level at different follow-up periods. AST: aspartate aminotransferase; PRP: platelet-rich plasma; SD: standard deviation.

Follow-up period	Mean (SD)		P-value
	Control	PRP	
After an hour	40.22 (38.07)	36.60 (40.81)	0.280
After 24 hours	47.00 (30.91)	27.47 (20.76)	0.119
After 7 days	56.77 (38.67)	36.14 (30.38)	0.161
After 14 days	31.00 (25.43)	29.95 (32.75)	0.161

## Discussion

Orthodontics stands out among dental specialties for its unique use of the inflammatory response to address both functional and aesthetic concerns. Recently, there has been a growing interest in methods to accelerate OTM. This interest is largely driven by patients’ desire for shorter treatment durations with orthodontic appliances [22]. Accelerated tooth movement techniques aim to reduce the overall time required for treatment, thereby enhancing patient satisfaction and compliance. These methods include both surgical and non-surgical approaches, each designed to expedite the remodeling of bone and the movement of teeth, ultimately achieving the desired dental alignment more efficiently [6].

Accelerated OTM has garnered significant attention due to its potential to reduce treatment duration, which is particularly advantageous for adult patients who experience slower tissue metabolism and are at higher risk for complications such as gingival inflammation and root resorption during prolonged treatment periods [23]. Various methods, including surgical techniques like corticotomy and piezocision, have been shown to effectively enhance OTM by activating local acceleratory phenomena, although these methods can be invasive [24]. However, the relationship between OTM and periodontal health remains complex. Orthodontic forces can induce inflammatory responses in the periodontium, which are necessary for tooth movement but may compromise oral hygiene and lead to increased bacterial formation, potentially exacerbating periodontal issues [25]. Therefore, while accelerated OTM can shorten treatment time and improve patient outcomes, careful management of periodontal health is essential to mitigate risks associated with orthodontic appliances [26].

The aim of this study was to evaluate the effects of PRP injections, a method of accelerating OTM, on the health of periodontal tissues. This method is less invasive and offers a gentler approach to accelerating tooth movement compared to surgical methods. Our results showed no statistically significant differences between the control group (conventional orthodontic treatment) and the PRP group in terms of PPD, CAL, BOP, and GR throughout the follow-up period. Both groups exhibited a slight increase in PPD, CAL, GR, and the percentage of BOP after four, eight, and 12 weeks compared to baseline. This is a normal outcome, as orthodontic treatment naturally induces gingivitis due to alterations in oral hygiene habits and periodontal health associated with the placement of orthodontic appliances.

On the other hand, AST enzyme levels increased after one hour, 24 hours, and seven days in the control group, with the highest value observed after seven days. However, 14 days later, the AST level decreased in the control group, with no statistically significant differences between the studied periods. Furthermore, there were no statistically significant differences between the two groups. This signifies the inflammatory process that normally occurs during orthodontic treatment. Our results indicate that there was no difference in AST enzyme levels between the two groups, asserting the safety of the PRP injection technique for accelerating OTM on periodontal tissues.

Our findings align with previous research that reported a significant increase in AST levels in GCF at both compression and tension sites on days seven and 14. This increase is likely due to controlled trauma, which induces cell death as a result of mechanical forces applied to the periodontal ligament (PDL) and alveolar bone [20]. Additionally, our study is comparable to another investigation that evaluated AST levels in GCF during canine distalization, where a similar increase was observed at the compression site [27]. The rise in AST levels indicates the application of orthodontic force on teeth and is directly associated with the compression site during OTM.

This study has a few limitations that should be considered. Firstly, the sample size was relatively small, which may affect the generalizability of the results. Additionally, the follow-up period was relatively short, so the long-term effects of PRP injections on periodontal health were not assessed. The study also focused on specific clinical parameters, and other potential indicators of periodontal health were not evaluated. Moreover, due to the nature of the interventions, it was not possible to blind patients, which may have introduced some bias. Future research with larger sample sizes, longer follow-up periods, and a broader range of clinical parameters would be beneficial to further validate and expand upon these findings.

## Conclusions

This study aimed to evaluate the effects of PRP injections on the health of periodontal tissues during accelerated OTM. Our findings indicate that there were no statistically significant differences between the control group (conventional orthodontic treatment) and the PRP group in terms of periodontal health parameters. Additionally, the AST enzyme levels did not show statistically significant differences between the two groups. Indicating that the PRP injection technique for accelerating OTM is safe for periodontal tissues.

Overall, our study supports the use of PRP injections as a less invasive and effective method for accelerating OTM without compromising periodontal health. Further research with larger sample sizes and longer follow-up periods is recommended to validate these findings and explore the long-term effects of PRP on periodontal tissues.
